# Temporal trends in mortality from pneumonia and obstructive sleep apnea in the United States (1999–2020): Insights from CDC WONDER

**DOI:** 10.1097/MD.0000000000047827

**Published:** 2026-02-20

**Authors:** Khawar Ali, Muhammad Talha, Muhammad Abdullah Mohsin, Syed Muhammad Salman Hassan, Kashf Younas, Zainab Kalsoom, Muhammad Ali, Muddassir Khalid

**Affiliations:** a Department of Medicine, Nishtar Medical University, Multan, Pakistan; b Department of Medicine, HCA Florida West Hospital, Pensacola, FL; c Department of Medicine, Bakhtawar Amin Medical and Dental College, Multan, Pakistan; d Department of Medicine, Fatima Jinnah Medical University, Lahore, Pakistan.

**Keywords:** adjusted mortality rate, pneumonia obstructive sleep apnea, mortality trends, CDC WONDER, age

## Abstract

Pneumonia is a significant cause of mortality in the United States. In the same way, obstructive sleep apnea (OSA) is a prevalent sleep-related breathing disorder that can disrupt the normal respiratory function through intermittent hypoxia, systemic inflammation, and impaired airway clearance. Data suggest that OSA may increase the chances of having pneumonia and can make the outcomes worse. To describe temporal trends and demographic patterns in mortality among US adults with pneumonia and coexisting OSA from 1999 to 2020. We performed a retrospective analysis by population using the Centers for Disease Control and Prevention Wide-Ranging Online Data for Epidemiologic Research multiple-cause-of-death database from 1999 to 2020. Pneumonia deaths were identified by International Classification of Diseases, Tenth Revision codes J09–J18, and OSA by International Classification of Diseases, Tenth Revision code G47.3. We have calculated age-adjusted mortality rates (AAMR) per 100,000 population. Joinpoint Regression Software was used to estimate annual percent change and average annual percent change with confidence intervals of 95%. This study complies with the Strengthening the Reporting of Observational Studies in Epidemiology guidelines. A total of 13,496 deaths from pneumonia and OSA in US adults aged ≥ 25 were identified during this study period—males exhibited higher mortality than females (AAMR: 0.38 vs 0.21). The total deaths in males were 8046, consisting 59.63% of the overall total deaths, while total deaths in females were 5450, consisting 40.38% of the overall total deaths. The AAMR rose from 0.09 in 1999 to 0.86 in 2020 (average annual percent change: 7.3307%, 95% confidence interval: 3.93–10.84). Between 1999 and 2020, mortality related to pneumonia among the patients with OSA increased significantly in the US, with significant demographic and geographic variations. There was a significant increase in death in male population 2018 onwards. These findings emphasize the need for better screening and management of OSA in populations that are at risk for pneumonia.

## 1. Introduction

Pneumonia is one of the major causes of death in the United States, especially among older adults and among those who have some comorbid conditions.^[1]^ In the same way, obstructive sleep apnea (OSA) affects a large proportion of adults. It is characterized by recurrent upper-airway collapse, intermittent hypoxia, sleep fragmentation, and increased activation of the sympathetic system. Sleep apnea is associated with chronic comorbidities and acute complications.^[2,3]^ Existing data shows that sleep apnea can cause an increased risk and severity of respiratory tract infections.^[3]^ These physiologic disturbances may affect immune defenses, promote inflammation and alter respiratory physiology, thus potentially increasing risk of having lower respiratory tract infections such as pneumonia. Studies suggest that OSA is linked with increased risk of pneumonia and more severe lower respiratory tract infections.^[4–6]^

Despite this, there is a lack of large-scale national data describing mortality trends in patients with both pneumonia and OSA.^[7,8]^ The increasing coexistence of pneumonia and OSA emphasizes the need to understand their relation to improve clinical outcomes and results. However, the patterns of pneumonia-related mortality among individuals having OSA have not been studied adequately.^[9]^ It is important to understand how mortality in this comorbid group has evolved over time for better public health planning and clinical prioritization. Given the population-level nature of mortality data available through the Centers for Disease Control and Prevention Wide-Ranging Online Data for Epidemiologic Research (CDC WONDER) database, a retrospective temporal trend design was employed. This approach allows for descriptive assessment of changes in age-adjusted mortality rates over time rather than causal inference regarding individual-level risk or treatment effects. Alternative observational designs requiring patient-level exposure data or matched comparison groups were not feasible within this publicly available dataset. In this study, we used the CDC WONDER database to evaluate and access the national mortality trends from 1999 to 2020 among US adults with Pneumonia and OSA, stratified by sex.^[10]^

## 2. Methods

### 2.1. Study setting and population

We have performed a population-based retrospective observational study. Death certificates were analyzed from the CDC WONDER public use database for the years 1999 to 2020 using International Classification of Diseases, Tenth Revision (ICD-10) codes. Codes used were J09–J18, G 47.3 for pneumonia, and OSA respectively. The multiple causes of death public records were used to analyze death certificates connected to pneumonia and OSA. All patients with pneumonia and OSA as the cause of death were included. All death records meeting the predefined ICD-10 criteria during the study period were included. No selection occurred at the level of testing, reporting, or clinical decision-making, as the analysis was based on complete national mortality files. This study has been exempted from obtaining clearance from the Institutional Review Board because it utilizes a de-identified public dataset made available by the government. It follows strict protocols of the government and helps protect the identity of the people who have died. This study is compliant with Strengthening the Reporting of Observational Studies in Epidemiology guidelines.

### 2.2. Case definition and data extraction

Data were gathered for demographics, place of death, year of death, urban/rural classification, regional split, and states. Information on race, ethnicity, age, and sex was determined by the demographics. The places of death included medical facility (inpatient, outpatient, and death on arrival), Decedent’s home, nursing home, hospice facility, unknown place of death, and others. Race/ethnicity was divided into the following categories based on information from death certificates and an earlier WONDER database study: Asian or Pacific Islander, White, Hispanic or Latino, and Non-Hispanics. Geographical regions were classified or categorized using the US Census Bureau’s guidelines into the Northeastern, Midwest, South, and Western areas. The National Center for Health Statistics Urban–Rural Classification Scheme has categorized the population into urban and rural areas based on the 2013 US census. Urban areas were further subdivided into a large central metro, a large fringe metro, a medium metro, and a small metro. We have applied the same classification scheme in this investigation. We included the deaths in which the contributing or underlying cause of death were pneumonia which was defined by ICD-10 codes J09–J18, and OSA, which was defined by ICD-10 code G47.3. CDC WONDER mortality data are derived from death certificates and are largely complete for age, sex, year, and cause of death. Cells with suppressed counts (<10 deaths) were excluded from subgroup analyses in accordance with CDC data-use guidelines. No imputation of missing values was performed.

### 2.3. Statistical analysis

Mortality data were analyzed per 100,000 using Joinpoint Regression Software. These rates were accompanied by 95% confidence intervals (CIs) and were broken down by sex, year, race, state, region, urban–rural status, and place of death. The total number of pneumonia and OSA-related deaths was divided by the matching US population for each year, thus getting the crude mortality rates. All mortality rates were age-standardized to the US standard population year 2000. The data on OSA-related Pneumonia were extracted from 1999 to 2020. We used the Joinpoint Regression Program (version 5.4.0, National Cancer Institute) to analyze the annual percent change (APC) and its related 95% CI in age-adjusted mortality rates (AAMR) to find any significant or major changes over time. No a priori pre- or post-intervention periods were defined. Instead, joinpoint regression was used to statistically identify inflection points in mortality trends. As a population-level observational analysis, the observed changes may reflect secular trends, evolving clinical practices, diagnostic awareness, coding practices, or external factors such as epidemics rather than discrete interventions. No multivariable adjustment for individual-level confounders was performed due to the aggregate nature of the data. Analyses assume that observed trends represent population-level associations rather than causal effects.

## 3. Results

Total 13,496 deaths occurred due to pneumonia and OSA from 1999 to 2020 among adults of United States aged ≥ 25 and older. Male total deaths were higher, that is, 8046 (59.6% of all deaths), as compared to female deaths, that is, 5450 (40.4%). Majority of deaths occurred in Inpatient-Medial facility numbering 10,114 (74.9%), followed by Decedent’s home 1185 (8.85%) and 1152 (8.5%) in Nursing Homes.

### 3.1. Descriptive mortality trends over time

**Figure 1.**
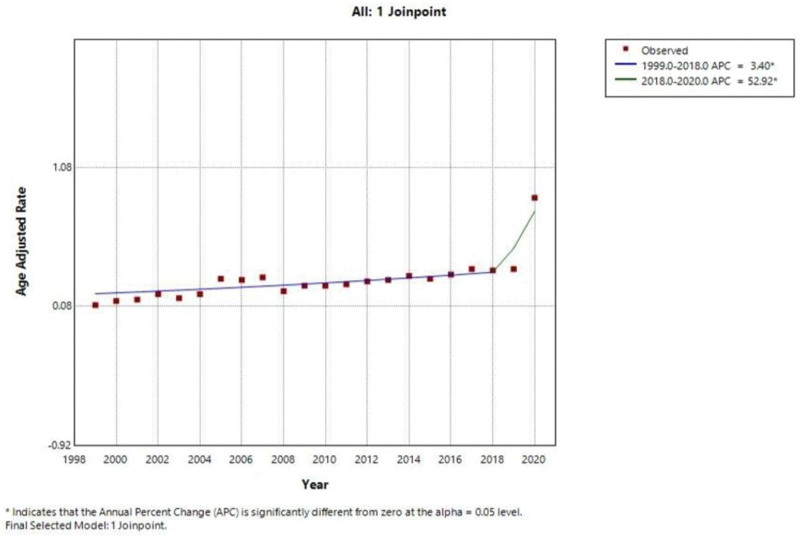
Overall annual mortality trends due to pneumonia and obstructive sleep apnea, 1999–2020.

The overall AAMR in patients with pneumonia and OSA was 0.29 (95% CI: 0.28–0.29) from 1999 to 2020. The AAMR increased from 0.09 in 1999 to 0.86 in 2020, corresponding to an average annual percent change (AAPC): 7.33% (95% CI: 3.93–10.84). Analysis by JoinPoint Regression Software showed an increase of AAMR from 1999 to 2018 at a steady rate with an APC of 3.404% (95% CI: 1.612–5.229). The overall AAMR then rose exponentially from 2018 to 2020 (APC: 52.920%; 95% CI: 10.638–111.361). A continuous upward trend with a steeper increase was observed after approximately 2018. These trends are illustrated here in Figure 1

### 3.2. Sex-stratified mortality patterns

**Figure 2.**
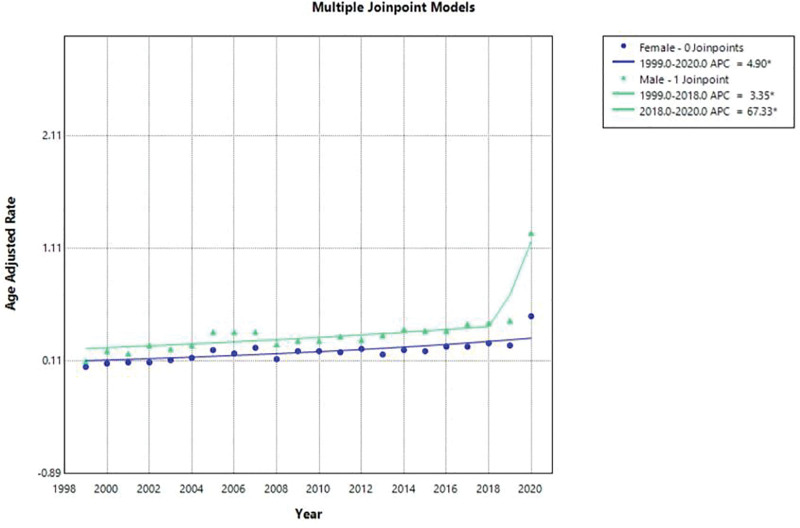
Sex-specific mortality trends due to pneumonia and obstructive sleep apnea, 1999–2020.

Between 1999 and 2020, the overall AAMR was invariably higher among males (AAMR: 0.38; 95% CI: 0.37–0.39) than in females (AAMR: 0.21% CI: 0.21–0.22). In 1999 there was male AAMR of 0.11 against female 0.06 (95% CI: 0.09–0.14 and 0.04–0.06), respectively. JoinPoint analysis demonstrated that the AAMR of males has been increasing steadily since 1999 to 2018 and the APC is 3.35% (95% CI: 1.473–5.263). This was followed by a sharp rise in rate between 2018 and 2020 with an APC of 67.325% (95% CI: 21.707–130.043) that was more than twice the former rate. The AAMR of females grew steadily in 1999 to 2020 with APC of 4.902% (95% CI: 2.957–6.884). AAPC in men was 8.2% (95% CI 4.78–11.73) and in women was 4.9% (95% CI 2.95–6.88), indicating that the increase is faster in men, particularly since 2018. Female did not indicate such spike provided 2018. These trends can be depicted in Figure 2.

### 3.3. Urbanization-stratified mortality patterns

**Figure 3.**
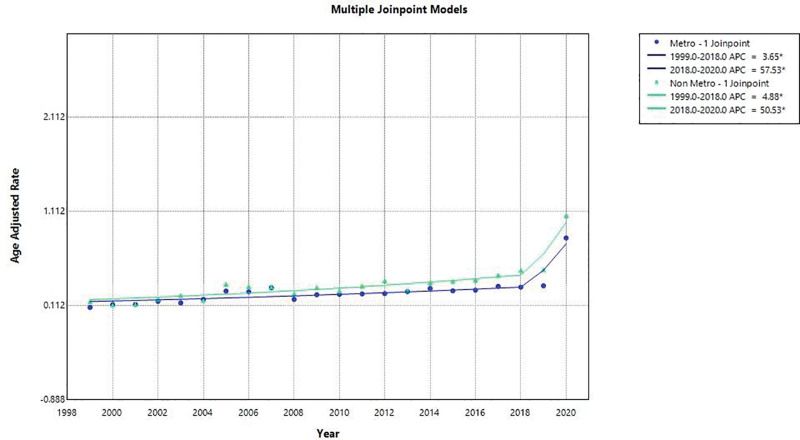
Urban–rural mortality trends due to pneumonia and obstructive sleep apnea, 1999–2020.

The overall AAMR of the residents of the metropolitan areas was higher (0.2863; 95% CI: 0.2806–0.2919), but not very strong, than the overall AAMR of the residents of nonmetropolitan areas. The JoinPoint analysis revealed that the number of patients living in the metropolitan regions had an increase in the number of AAMR between 1999 and 2018 with an APC of 3.65 (95% CI: 1.843–5.491) and then a sharp increase in the number between 2018 and 2020 with an APC of 57.53% (95% CI: 11.916–121.738). It was found that the AACP in urban locations of metropolitan areas in the study area is 7.862% (95% CI: 4.289–11.55). The AAMR of the rise in the Nonmetropolitan residents was continuously rising during the period of 1999 to 2018 with the APC of 4.88% (95% CI: 3.034–6.766). Subsequently, it experienced a steep increase between 2018 and 2020 with an APC value of 50.53% (95% CI: 8.199–109.43). In the nonmetropolitan areas, the AAMR grew at a rate of 8.556% (95% CI: 5.050–12.178) over the period. These results are shown in Figure 3.

### 3.4. Geographical regions-stratified mortality patterns

**Figure 4.**
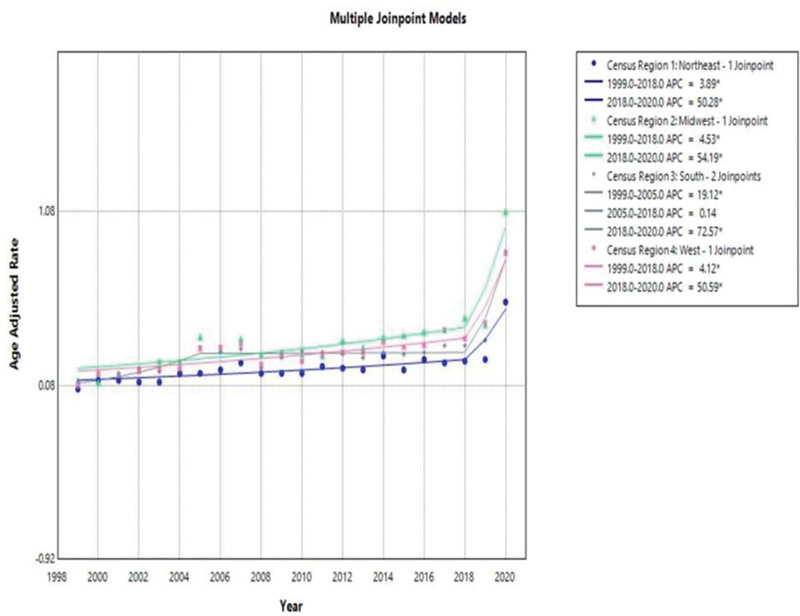
Geographic mortality trends due to pneumonia and obstructive sleep apnea, 1999–2020.

The overall AAMR of the residents of the Midwest was the largest (stratified by the Unites States census regions) with 0.35 (95% CI: 0.34–0.36), followed by the West (AAMR: 0.32; 95% CI: 0.31–0.33), South (AAMR: 0.29; 95% CI: 0.28–0.3), and lastly North east (AAMR: 0 JoinPoint analysis indicated that Midwestern residents have recorded the highest increase in the period between 1999 and 2020, at APC of AAPC 8.4763% (4.49–12.61). The same pattern was followed by West, and the sharp increase was achieved, resulting in an AAPC of 7.84% (95% CI: 4.31–11.49). It occurred similarly among the population of Northeast, and there was a significant mortality increase, as the AACP is 7.60 with a 95% confidence interval of 3.9 to 11.37. Finally, the community of South had an acute yet slightly uneven rise in AAMR between 1999 and 2020, and the AAPC is 10.83% (95% CI: 6.85–11.94). In 1999 to 2018, the APC of Midwest was 4.534 (95% CI: 2.518–6.59) and 54.187 (95% CI: 5.43–125.496). West was 4. 119 (95% CI: 2.33–5.94) in 1999 to 2018 and 50.59 (95% CI: 7.38–111.184). The South APC of 1999 to 2005 = 19.12 (95% CI: 7.065–32.53) compared to the 2005 to 2018 = 0.142 (95% CI: 1.932–2.26) and was 72.574 (95% CI: 36.55–188.096). Finally, Northeast APC was 3.89 (95% CI 1.99–5.82) in the year 1999 to 2018 and 50.28 (95% CI: 6.155–122.76) in 2018 to 2020. The findings are presented in Figure 4.

### 3.5. Race-stratified mortality patterns

When stratified by the US racial groups with the reliable data, we found that White had a steady increase throughout the years while Black had a spike from 2018 onwards. AAMR for Black was 0.32 (95% CI: 0.3–0.33) and for White it was 0.29 (95% CI: 0.28–0.29). White has single APC from 1999 to 2000, that is, 5.74 (95% CI 3.64–7.88). In case of Black APC from 1999 to 2018 was 2.042 (95% CI: 0.081–4.107) and from 2018 to 2020 it was 87.37 (95% CI: 25.48–179.78). AAPC for Black was 8.122% (95% CI 3.96–12.45). The data for other groups was unreliable.

**Figure 5.**
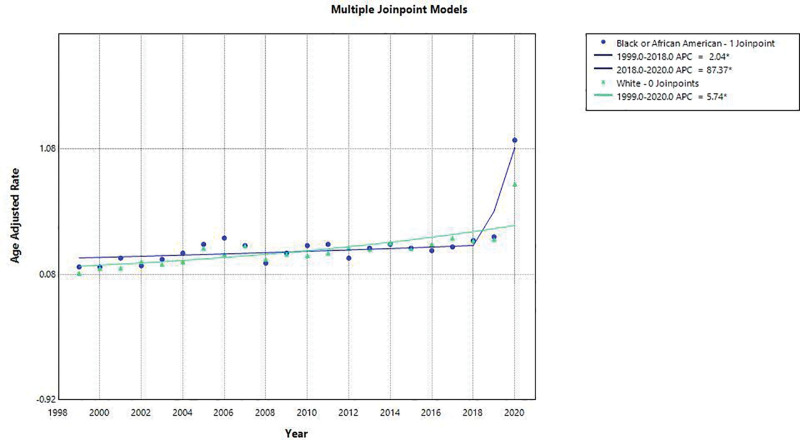
Racial and ethnic mortality trends due to pneumonia and obstructive sleep apnea, 1999–2020.

The results are depicted in Figure 5.

## 4. Discussion

This is a nationwide study on the United States in 1999 and 2020, which indicates that mortality in adults with pneumonia and OSA considerably increased. These 22 years had more than 13,496 total deaths with the males always recording more death as compared to the females. They build on other previous studies that indicate that OSA is linked to a greater risk of pneumonia and worse respiratory infections. The findings we got offer valuable insights into the management of both pneumonia and OSA carrying significant implications for clinical practice. Over the past 2 decades, there has been a significant increase in overall mortality rates related to pneumonia and OSA. However, the increase in mortality among males is more significant than among females especially after 2018.

Our study observed a steady increase in the overall AAMR from 1999 to 2018, followed by a significant spike in 2018 to 2020.

Several mechanisms may underlie the observed trends. First, OSA-associated sleep fragmentation, intermittent hypoxia, impaired cough reflex and increased risk of aspiration may contribute to more severe pneumonia.^[11,12]^ Intermittent hypoxia can affect immune cell function, reduce mucociliary clearance, and increase susceptibility to both bacterial and viral pathogens. Second, raised obesity rates, aging populations and greater recognition of OSA may cause an increase in the pool of individuals at risk.^[13–16]^ Third, although OSA diagnosis and treatment have improved, data suggest that many remain untreated or undiagnosed; thus, the burden of untreated OSA may contribute to higher mortality when pneumonia occurs.^[13,17,18]^ In addition, the mechanical effects of obstructive events promote microaspiration of oropharyngeal secretions during sleep, which may introduce pathogens into the lower respiratory tract. These mechanisms provide biological plausibility for the higher pneumonia-related mortality observed in OSA patients.^[19]^

Our findings have important implications. They suggest the need for heightened awareness of OSA as a comorbidity in patients presenting with pneumonia, particularly older males. Early screening and management of OSA, along with preventive measures (e.g., vaccination, weight management, and aspiration risk reduction), may help reduce mortality in this high-risk group.^[20,21]^

Our findings are consistent with prior clinical and epidemiological research demonstrating a link between OSA and poor respiratory outcomes. Smith et al reported that OSA patients had higher rates of community-acquired pneumonia and longer hospital stays compared with controls. Similarly found that OSA severity correlated with worse outcomes in viral pneumonia, including COVID-19, emphasizing that hypoxia-driven immune dysregulation may worsen infectious respiratory illnesses.^[22]^ Recent data based on population have also showed that OSA predicts independently the increased hospital readmission and mortality related to pneumonia, even after adjustment for age, sex, and comorbidities.^[23]^ These collective findings emphasize that OSA is not only a sleep-related disorder but also a systemic condition that has an impact of infection susceptibility and recovery. Our population-level findings extend these prior observational studies by demonstrating consistent national mortality trends over more than 2 decades.

Demographic trends in our data are related to the general population, with higher mortality among males. OSA severity is greater in men, and this is consistent with prior reports, so male sex is a risk factor for both pneumonia and sleep-disordered breathing.^[24]^

By adding geographical regions as a variable, we found substantial regional heterogeneity in patterns of mortality. The highest mortality burden was found in the Midwest, followed by the West, which may be related to higher obesity prevalence, greater OSA burden, and regional variations in healthcare facilities. Conversely, the Southern and Northeast regions showed relatively lower mortality trends. Such disparities can also suggest the difference in socioeconomic factors, vaccination, and access to specialized sleep medicine. The methods to comprehend these regional trends in detail are very essential because they imply that the local health care interventions must be region-specific and need-based in relation to the resources in its disposal.

Urban–rural stratification revealed that the mortality rate of OSA patients with pneumonia was more always low in the metropolitans setting compared to the Nonmetropolitan environment. The Nonmetropolitan populations often have lower access to sleep specialists, sleep labs, pulmonary care, and emergency timely services. In addition, the risk could be aggravated by the fact that rural communities are more prone to higher rates of obesity, smoking, and untreated comorbidities. In this case too, limited access to the continuous positive airway pressure (CPAP) equipment, weak follow-up, and health literacy might also play a role. These findings highlight the importance of developing rural healthcare infrastructure and implementing telemedicine-based sleep care services to fill the gap in diagnosis and management.

There are various important implications in our study. The observed increase in mortality due to Pneumonia among individuals with OSA highlights the ultimate need for increased special clinical care and vigilance in this high-risk group. Early diagnosis of respiratory infections, optimization of OSA management, and education of patients related to CPAP hygiene can contribute to the reduction of complications. Moreover, pneumonia risk in OSA populations could be decreased indirectly by public health programs targeting cardiometabolic health and obesity. Research integrating clinical, microbiological, and polysomnographic data is warranted for the clarification of causative mechanisms and to discover whether specific OSA phenotypes confer greater infection risk.

Analysis of place of death provided additional information about care utilization at end-of-life. Most deaths of OSA patients related to pneumonia were in the hospital although a progressive percentage of pneumonia deaths were at home or in the long-term care facility during the study period. This can reflect changes in chronic disease care, a rising prevalence of nonhospital end-of-life care or decreased access to acute care especially among the elderly or rural population. The increased mortality rates in the long-term care facilities can also be associated with the poor identification of sleep-disordered breathing, under-optimal adherence to CPAP treatment, and the elevated risk of respiratory infections in the congregate facilities. These results highlight the necessity of the enhanced OSA screening and CPAP management procedures in nursing facilities and residential care conditions.

The added racial and ethnic variables also demonstrated significant inequalities, particularly with the Blacks having higher age-adjusted mortality rates than the Whites, namely, mainly in the recent years, there is a sudden rise in deaths among Black due to OSA-related pneumonia. Earlier studies have also documented that minority groups have challenges to early diagnosis and effective treatment of OSA such as poor completion of sleep study and poor adherence to CPAP.^[25]^ Accordingly, racial differences in the recent years that were observed in our data point towards the necessity of culturally appropriate screening approaches and equal access to sleep and respiratory treatment.

Combined with these other demographic and contextual factors, these further demographic and contextual variables enrich the idea that the connection between pneumonia and OSA is planned by an intricate connection of age, race, geography, and healthcare access. The disparity observed in these groups underscores the fact that OSA-related pneumonia mortality is not a clinical problem but also a public health and health-equity concern.

The disparity observed among these groups indicates that pneumonia attributed to the use of OSA needs to be considered not only a clinical problem but also a health-equity and a public health concern. The various solutions to these multidimensional variables, which include enhancing access to diagnostic sleep studies, development of CPAP support programs, and enhancing public health outreach in underserved communities can potentially lead to a significant decrease in mortality in at-risk populations.

### 4.1. Limitations

As a before-after-type temporal trend analysis, this study is subject to temporal confounding, including changes in diagnostic coding, healthcare access, treatment practices, and population health characteristics over time. Regression to the mean and secular trends cannot be excluded. Our study relies on death certificate data and ICD-10 coding, due to which our diagnoses can be misclassified. There was a lack of data on OSA severity, treatment (e.g., CPAP use), obesity, smoking status, and other potential confounders. Further, causality cannot be inferred. But still, long-time spans and large national samples strengthen the validity of the observed temporal trends.

## 5. Conclusion

In the United States between 1999 and 2020, there is significant increase in mortality among adults with pneumonia and coexisting OSA, with male showing largest increase especially after 2018. These findings highlight an association between coexisting OSA and increasing pneumonia-related mortality, underscoring the importance of integrating sleep-disordered breathing evaluation and management of care pathways and public health strategies for pneumonia.

## Author contributions

**Conceptualization:** Khawar Ali, Syed Muhammad Salman Hassan.

**Formal analysis:** Zainab Kalsoom.

**Methodology:** Kashf Younas.

**Project administration:** Muddassir Khalid.

**Software:** Muhammad Ali.

**Supervision:** Muddassir Khalid.

**Validation:** Muhammad Abdullah Mohsin.

**Visualization:** Muhammad Abdullah Mohsin.

**Writing – original draft:** Khawar Ali.

**Writing – review & editing:** Muhammad Talha, Muddassir Khalid.
